# RNA-Seq-Based Transcriptome Analysis of Chinese Cordyceps Aqueous Extracts Protective Effect against Adriamycin-Induced mpc5 Cell Injury

**DOI:** 10.3390/ijms251910352

**Published:** 2024-09-26

**Authors:** Hailin Long, Mengzhen Liu, Zhongchen Rao, Shanyue Guan, Xiaotian Chen, Xiaoting Huang, Li Cao, Richou Han

**Affiliations:** 1 Guangdong Key Laboratory of Animal Conservation and Resource Utilization, Guangdong Public Laboratory of Wild Animal Conservation and Utilization, Institute of Zoology, Guangdong Academy of Sciences, Guangzhou 510260, China; hlin_long@163.com (H.L.); 15249697542@163.com (M.L.); xipredus@163.com (Z.R.); huangxt1001@163.com (X.H.); caol@giz.gd.cn (L.C.); 2 Guangdong Provincial Key Laboratory of Insect Developmental Biology and Applied Technology, Institute of Insect Science and Technology, School of Life Sciences, South China Normal University, Guangzhou 510631, China; 3 Instrumental Analysis and Research Center, Sun Yat-sen University, Guangzhou 510275, China; pusgshy@mail.sysu.edu.cn; 4 Center for Industrial Analysis and Testing, Guangdong Academy of Sciences, Guangzhou 510650, China; chenxiaotian2012@163.com

**Keywords:** Chinese cordyceps aqueous extracts, podocyte injury, RNA-seq, molecular mechanism, nephrotic syndrome, qRT-PCR

## Abstract

Pharmacogenomic analysis based on drug transcriptome characteristics is widely used to identify mechanisms of action. The purpose of this study was to elucidate the molecular mechanism of protective effect against adriamycin (ADM)-induced mpc5 cell injury of Chinese cordyceps aqueous extracts (WCCs) by a systematic transcriptomic analysis. The phytochemicals of WCCs were analyzed via the “phenol–sulfuric acid method”, high-performance liquid chromatography (HPLC), and HPLC–mass spectrometry (MS). We analyzed the drug-reaction transcriptome profiles of mpc5 cell after treating them with WCCs. RNA-seq analysis revealed that WCCs alleviated ADM-induced mpc5 cell injury via restoring the expression of certain genes to normal level mainly in the one-carbon pool by the folate pathway, followed by the relaxin, apelin, PI3K-Akt, and nucleotide-binding, oligomerization domain (NOD)-like receptor signaling pathway, enhancing DNA synthesis and repair, cell proliferation, fibrosis reduction, and immune regulation. Otherwise, WCCs also modulated the proliferation and survival of the mpc5 cell by regulating metabolic pathways, and partially restores the expression of genes related to human disease pathways. These findings provide an innovative understanding of the molecular mechanism of the protective effect of WCCs on ADM-induced mpc5 cell injury at the molecular transcription level, and *Mthfd2*, *Dhfr*, *Atf4*, *Creb5*, *Apln*, and *Serpine1*, etc., may be potential novel targets for treating nephrotic syndrome.

## 1. Introduction

Nephrotic syndrome is one of the well-known presentations of pediatric and adult renal disease. This disease predominantly prefers men more than women, and the most common causes of nephrotic syndrome are diabetic nephropathy, focal segmental glomerulosclerosis, and membranous nephropathy [1,2]. Nephrotic syndrome, which is characterized by the presence of proteinuria, edema, hypoalbuminemia, and hyperlipidemia, is a disorder of the glomerular filtration barrier in the nephron that leads to massive protein and fluid loss through the kidneys [3,4]. The glomerular filtration barrier consists of three structures: glomerular endothelial fenestrae, glomerular basement membrane (GBM), and the podocyte interfoot process. Podocyte are located at the outermost side of the GBM and are the last and most important structural layer of the glomerular filtration barrier [5,6,7]. Therefore, podocyte protection is of considerable significance to prevent or delay the process of nephrotic syndrome.

Traditional Chinese medicine (TCM) has been used for thousands of years and has been proven to be effective at treating many complicated illnesses with minimal side effects [8,9]. The Chinese cordyceps (CC), a unique parasitic complex of mummified *Thitarodes/Hepialus* spp. (Hepialidae, Lepidoptera) cadaver by *Ophiocordyceps sinensis* fungus in the Qinghai-Tibet Plateau, is one of the most well-known TCMs in Asian countries since the 15th century [10,11]. The traditional usage of this cordyceps was recorded in a book in the Qing Dynasty (1757 AD) [12]. It is usually supplied to sub-healthy and sick people [13]. TCM usually comprises hundreds of different constituents with widely differing physiochemical properties [14,15]. The main chemical ingredients of CC are sterols, higher-fatty acids, nucleosides, and polysaccharide, which were reported in our previous study [16]. CC is reported to possess various biological activities, including anti-aging, antioxidant, reparative, anti-cancer, immune-stimulation, and renal-protective properties [17,18,19]. It has been widely used to treat chronic kidney disease including diabetic nephropathy, IgA nephropathy, lupus nephritis, renal transplant recipients, and nephrotic syndrome [20,21,22,23]. However, the protective effect against podocyte injury and molecular mechanism of Chinese cordyceps have not been thoroughly investigated.

Pharmacological perturbations by drugs (chemical synthesis and natural sources) that bind to targets stimulate cellular signal transduction and induce particular gene expression patterns [24]. Therefore, transcriptome properties are considered an ideal choice for identifying drug modes of action and molecular mechanisms [25]. Due to the presence of multiple components in TCM, they have the advantage of having a varieties of effects on various disease targets [26]. However, an integrated representation of the molecular mechanisms and downstream targets of TCM extracts and related phytochemicals is still lacking. Moreover, due to the synergistic or antagonistic effects between components, the overall molecular effects of TCM may differ from the sum of the effects of individual components of TCM. In addition, even the same herb may have different medicinal effects when using different extraction methods. Therefore, it is necessary to systematically generate and analyze transcriptome data of TCM.

The core of our study was to further explore whether WCCs have podocyte protective activity as well as the potential for the treatment of nephrotic syndrome, and then to search for its possible molecular mechanism. Here, we generated the drug-responsive transcriptome data for CC, which were tested on the mpc5 mouse renal podocyte cell line with multiple doses and extraction methods. Systematic transcriptome analysis revealed the molecular mechanism associated with the protective effect of WCCs against ADM-induced podocyte injury and highlighted the differences between the effects of different Chinese cordyceps extracts. Our study indicated that comprehensive analysis using drug response transcriptome data can provide a deeper understanding of the molecular mechanisms of TCM.

## 2. Results

### 2.1. Polysaccharides Content of WCCs

The aqueous extracts were the main bioactivity ingredients of Chinese cordyceps, accounting for more than 90% of the weight ratio, mainly including proteins, polysaccharides, adenosine, and nucleosides. W1CC, W2CC, W3CC, and W4CC were precipitates formed by sequentially adding ethanol to aqueous extract of Chinese cordyceps at concentrations of 20%, 40%, 60%, and 80%, mainly containing polysaccharides. W5CC was the remaining ingredients of aqueous extract of Chinese cordyceps after removing the precipitates. The phenol–sulfuric acid method was used to determine the content of polysaccharides in W1CC, W2CC, W3CC, W4CC, and W5CC, and the results showed that the polysaccharide content were 49%, 25%, 11%, 10%, and 7%, respectively.

### 2.2. Mainly Nucleoside Ingredients in W5CC

**Figure 1:**
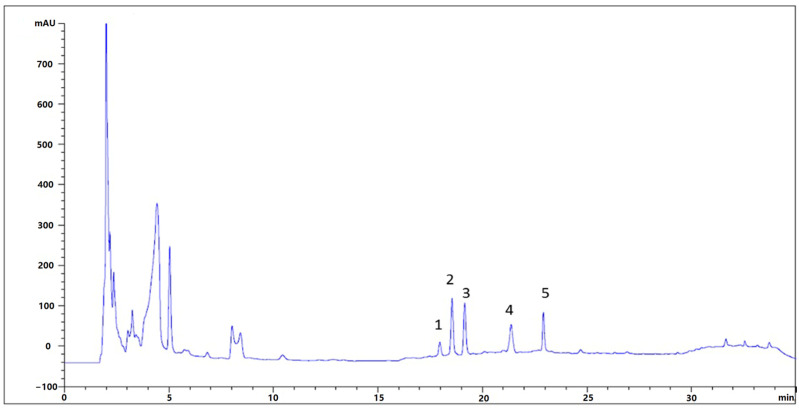
The HPLC fingerprint of W5CC (peak1: inosine; peak2: guanosine; peak3: 2′,3′-dideoxyxanthosine; peak4: thymine; peak5: adenosine).

W5CC mainly contained nucleoside ingredients such as adenosine, inosine, and guanosine. By comparing with the retention time of reference substances and mass spectral data in the available literature, some chromatographic peaks in the HPLC fingerprint were identified, including peak1, inosine; peak2, guanosine; peak3, 2′,3′-dideoxyxanthosine; peak4, thymine; and peak5, adenosine (Figure 1).

### 2.3. ADM-Induced Podocyte Injury Cell Model

**Figure 2:**
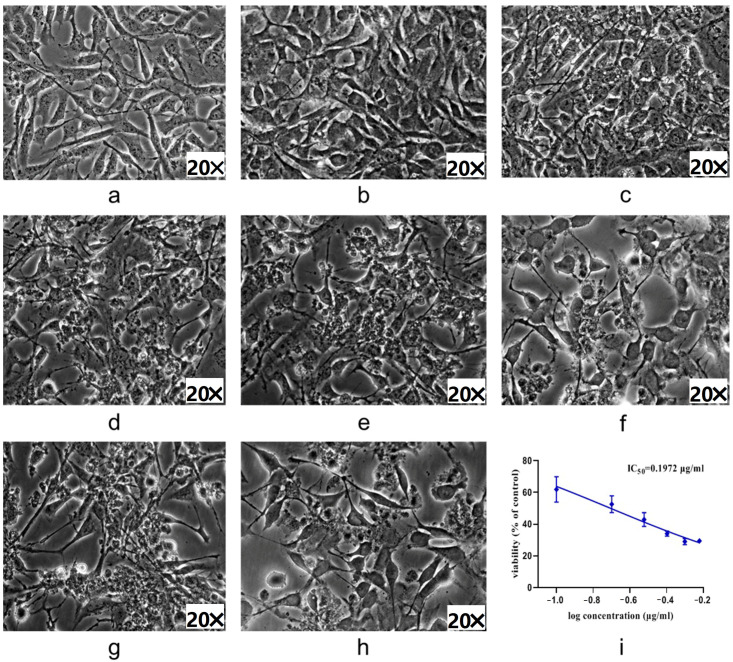
Growth morphology of podocyte induced by different concentrations of ADM ((**a**) NC × o h; (**b**) NC × 24 h; (**c**) 0.1 μg/mL ADM × 24 h; (**d**) 0.2 μg/mL ADM × 24 h; (**e**) 0.3 μg/mL ADM × 24 h; (**f**) 0.4 μg/mL ADM × 24 h; (**g**) 0.5 μg/mL ADM × 24 h; (**h**) 0.6 μg/mL ADM × 24 h; (**i**) IC_50_ profile).

Normal podocytes were polygonal or spindle-shaped, with clear cell contours and a small amount of protrusions extending from the cell body (Figure 2a), and the cells appeared as cobblestones when growing to a fully fused state (Figure 2b). Induced with various concentrations of ADM for 24 h, the morphology of mpc5 cells showed no significant change at 0.1 μg/mL, but the number of cells slightly decreased (Figure 2c). Cell growth was inhibited, and the morphology changed significantly after exposure to 0.2 μg/mL or 0.3 μg/mL ADM for 24 h. The foot process of mpc5 cells began to retract and a small number of cells appeared as cobblestones (Figure 2d,e). Cell numbers further decreased, and the foot process fusion and retraction were more obvious when exposed at 0.4 μg/mL ADM for 24 h (Figure 2f). The cells were basically apoptotic, and the residual cells exhibited a cobblestone appearance after exposed at 0.5 μg/mL or 0.6 μg/mL ADM for 24 h (Figure 2g,h). The IC_50_ value of viability was 0.1972 μg/mL (Figure 2i). Therefore, the ADR-induced nephropathy model was established by inducing mpc5 cell with 0.20 μg/mL ADM for 24 h.

### 2.4. Effect of WCCs on mpc5 Cell Viability

**Figure 3:**
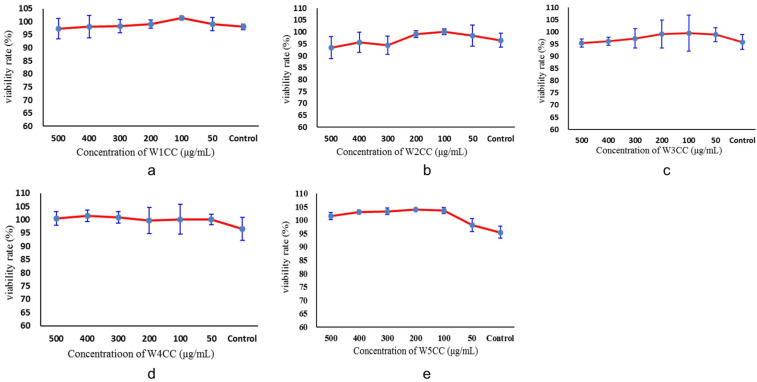
Cytotoxicity of WCCs on mpc5 cell viability ((**a**): W1CC; (**b**): W2CC; (**c**): W3CC; (**d**): W4CC; (**e**): W5CC; data are presented as mean ± SD (n = 3)).

The cytotoxicity assay showed that W1CC, W2CC, W3CC, W4CC, and W5CC had no significant inhibitory effect on mpc5 cell even at the concentration of 500 μg/mL (Figure 3). Based on these results, the following concentrations were selected for mpc5 protective activity assay: 100 μg/mL, 300 μg/mL, and 500 μg/mL for W1CC, W2CC, W3CC, W4CC, and W5CC.

### 2.5. Protective Efficacy of WCCs on ADM-Induced mpc5 Cell

**Figure 4:**
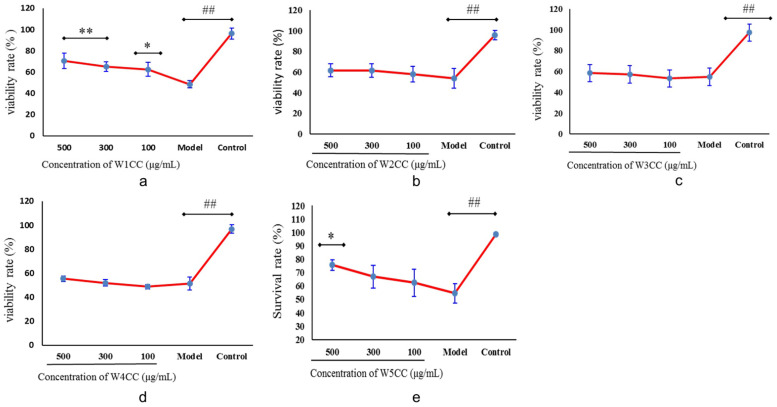
Protective efficacy against ADM-induced mpc5 cell injury of WCCs ((**a**): W1CC; (**b**): W2CC; (**c**): W3CC; (**d**): W4CC; (**e**): W5CC; data are presented as mean ± SD (n = 3); ^##^ indicates *p* < 0.01 as compared with control group; * indicates *p* < 0.05 or ** indicates *p* < 0.01 as compared with model group).

The bioactivity assay showed that W1CC at the concentrations of 100, 300, and 500 μg/mL played an important role in promoting proliferation (Figure 4a) and cell morphological maintenance effect for ADM-induced mpc5 cell injury model, and W5CC at 500 μg/mL also exerted the same bioactivity (Figure 4e). W2CC, W3CC, and W4CC did not exhibit stable bioactivity in ADM-induced mpc5 cell injury model at 100 μg/mL, 300 μg/mL, or 500 μg/mL (Figure 4b–d).

### 2.6. WCCs Alter RNA Expression Profiles in mpc5 Cell

Whole-mRNA sequencing was used to monitor changes in gene expression among mpc5 cell treatments with ADM, W1CC, and W5CC. In total, 44 Gb of high-quality clean data was obtained from twelve libraries, including six libraries of W1CC and W5CC, three normal control libraries (NC), and three model control libraries (ADM), with at least 95.84% clean reads matching the *Mus musculus* annotated genome (Genome Reference Consortium, 2020; GCF_000001635.27) using HISAT (2.0.6) (Table 1). The FPKM value of expressed genes was shown in Table S1. The correlation coefficient between the biological replicated samples of the same treatment always exceeded 0.98, which was higher than the values between the different treatment samples.

To gain insights into global transcriptional changes in mpc5 cells induced by ADM damage, pairwise comparisons were performed between the ADM and NC libraries to identify differentially expressed genes (DEGs). A total of 6038 DEGs were obtained (|log_2_FC| ≥ 0.5 and false discovery rate (FDR) < 0.05), including 2691 and 3347 DEGs’ up- and downregulation, respectively. A complete list of DEGs was provided in Table S2.

**Figure 5:**
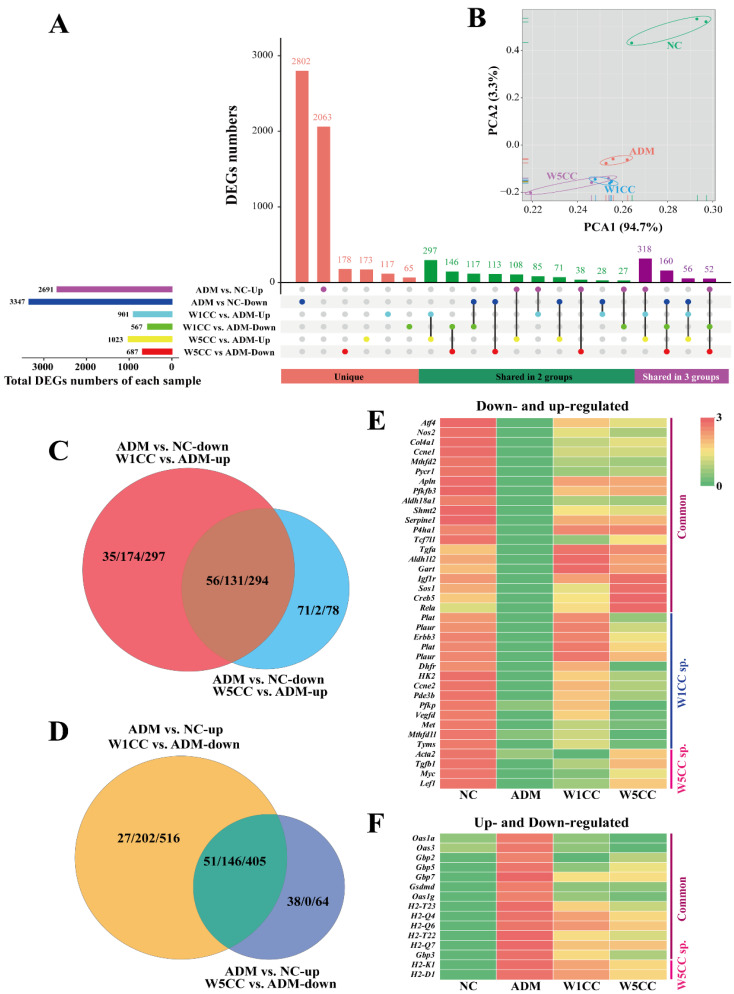
Differentially expressed gene analysis: (**A**) UpSetR plots depict unique and shared DEGs among different comparing groups. Horizontal bars represent the total DEG numbers of each comparing group; vertical bars represent the unique and shared DEGs of different comparing group intersections. (**B**) Principal component analysis (PCA) of the transcriptome for different treatments. Different treatments are shown in different colors. Venn analysis of the up-regulatory (**C**) and down-regulatory (**D**) effects of W1CC and W5CC treatment after mpc5 cell were injury by ADM, whereas the heatmap (**E**,**F**) provides a global view of the most important DEGs involved in protective effect against ADM-induced mpc5 cell injury. Three numbers among two slashes mean level 1 genes/level 2 genes/level 3 genes. Detailed information on gene levels is presented in the Section 4. NC: normal control; ADM: treated with ADM; W1CC: treated with ADM and W1CC; W5CC: treated with ADM and W5CC.

To explore the molecular mechanism of protective effect against ADM-induced mpc5 cell injury of WCCs, the DEGs were identified between sample groups (W1CC and W5CC) and the ADM group. In comparing W1CC and ADM, 1468 DEGs were identified, among which 901 were upregulated and 567 were downregulated. Subsequently, a pairwise comparison was conducted between W5CC and ADM, identifying 1710 DEGs, including 1023 upregulated and 687 downregulated genes (Figure 5A).

Mpc5 cells stimulated by ADM exhibited alterations in gene expression levels, ultimately leading to specific injury patterns in the mpc5 cells. The treatment of W1CC and W5CC promoted the downregulation of genes overexpressed, while simultaneously upregulating the genes previously suppressed by ADM in the mpc5 cells. Principal component analysis (PCA) was also performed for the DGEs (Figure 5B). Simultaneous comparison of NC, ADM, and sample groups (W1CC and W5CC) were performed. Genes that were up-/downregulated following ADM stimulation and returned to near-normal after being treated with W1CC or W5CC were employed for further analysis (level 1 and level 2 genes). Furthermore, genes that remained unaltered after ADM treatment but exhibited specific up- or downregulation subsequent to W1CC or W5CC treatment were also taken into account (level 3 genes). Detailed information was listed in Tables S3 and S4. Venn analysis was also conducted for the three levels of genes. The genes sharing between the W1CC and W5CC groups, indicating the same mechanisms were employed by both the W1CC and W5CC in alleviating mpc5 cell injury induced by ADM (Figure 5C,D). The most important genes involved in protective effect against ADM-induced mpc5 cell injury were further detailed by plotting the DEGs on a heatmap (Figure 5E,F).

### 2.7. Enrichment Analysis

**Figure 6:**
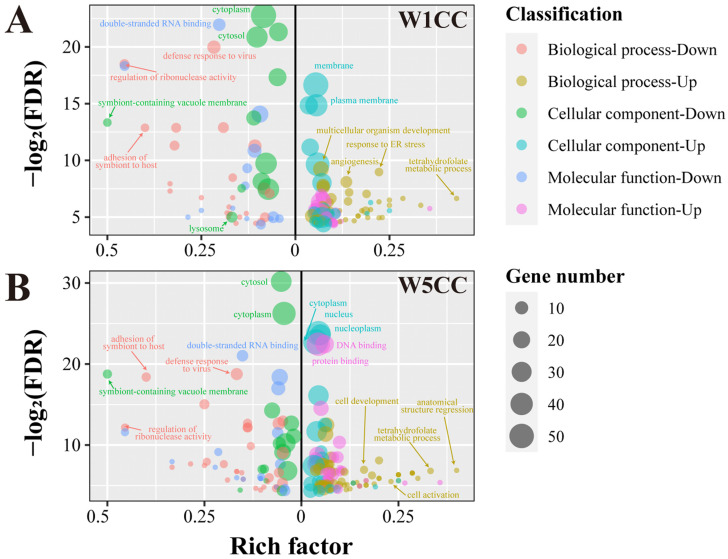
GO enrichment analysis for the call-back genes after W1CC (**A**) and W5CC (**B**) treatments. The right and left segments of the X-axis denote the rich factor of different GO terms with up- and downregulatory effects, respectively. Biological processes, cellular components, and molecular functions with different call-back effects are assigned to different colors, and the size of the bubble was indicative of the gene number. Biological process-up (-down) correspond to genes in biological process’ GO terms with up (down) call-back effects (log_2_FC_ADM vs. NC_ < −0.5 (>0.5), log_2_FC_W1CC/W5CC vs. ADM_ > 0.5 (<−0.5), FDR < 0.05), with others conforming accordingly. ADM; treated with ADM; W1CC: treated with ADM and W1CC; W5CC: treated with ADM and W5CC.

**Figure 7:**
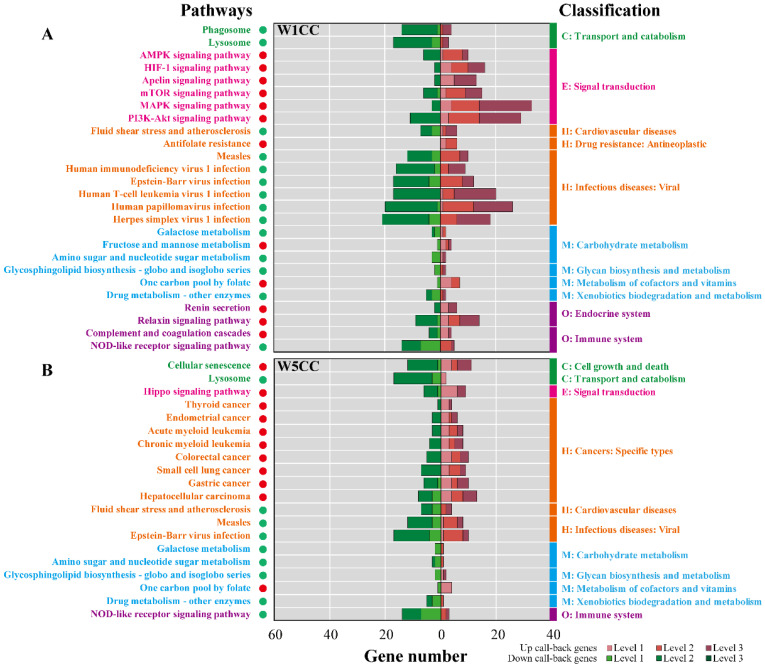
Pathway enrichment analysis for the call-back genes after W1CC (**A**) and W5CC (**B**) treatments. The Y-axis represented the pathways, while the right and left segments of the X-axis (divided by “0”) denoted the gene number of different pathways with up- and downregulatory effects, respectively. The pathways significantly enriched by up/downregulated genes were represented by a “red/green” circle, respectively. Pathways in different classifications were indicated by different colors. Level 1 genes were significantly down-/upregulated (|log_2_FC| > 0.5, FDR < 0.05) after ADM treatment, meanwhile significantly up-/downregulated after W1CC/W5CC treatments. Level 2 genes were significantly or slightly down-/upregulated after ADM treatment, meanwhile significantly or slightly up-/downregulated after W1CC/W5CC treatments. Level 3 genes were not changed after ADM treatment, but significantly or slightly up-/downregulated (|log_2_FC| > 0, FDR < 0.05) after W1CC/W5CC treatments. ADM: treated with ADM; W1CC: treated with ADM and W1CC; W5CC: treated with ADM and W5CC.

For the W1CC upregulated genes, level 1, 2, and 3 genes were assigned to 437, 651, and 1180 Gene Ontology (GO) processes, respectively. For the level 1 genes, angiogenesis, apoptotic process, one-carbon metabolic process, regulation of cell population proliferation, multicellular organism development, tetrahydrofolate metabolic process, and response to ER stress in the biological process were significantly enriched, mainly located in the membrane, extracellular space, and cytoplasm, leading the molecular function of catalytic activity and binding (Figure 6A). Pathway enrichment analysis was also performed. The corresponding genes were assigned to 30 (level 1), 93 (level 2), and 121 (level 3) pathways, respectively. One carbon pool by folate was the most significantly enriched pathway for both level 1 and level 2 genes, followed by the apelin, HIF-1, MAPK, and PI3K-Akt signaling pathways from the signal transduction, renin secretion, and relaxin signaling pathway from the endocrine system (Figure 7A). On the other hand, the W1CC downregulated genes were assigned to 360 (level 1), 496 (level 2), and 976 (level 3) GO processes. For the level 1 genes, defense response to virus, regulation of ribonuclease activity, adhesion of symbiont to host, and immune system process in biological processes were significantly enriched. For the pathway enrichment analysis, the downregulated genes in W1CC were enriched in 32 (level 1), 120 (level 2), and 128 (level 3) pathways, respectively. The NOD-like receptor signaling pathway from the immune system, lysosome and phagosome from transport and catabolism pathways, amino sugar and nucleotide sugar, drug, and galactose metabolism, and several pathways from viral infectious diseases such as measles were significantly enriched for level 1 and level 2 genes. Detailed information on GO and pathway enrichment analysis of level 1, 2, and 3 genes is listed in Tables S5 and S6.

For the W5CC upregulated genes, level 1, 2, and 3 genes were assigned to 529, 717, and 869 GO processes, respectively. The level 1 genes were significantly enriched in the biological processes such as regulation of transcription by RNA polymerase II, cell migration, cell development, and cell activation; cellular components such as nucleus, nucleoplasm, and cytoplasm; and molecular functions such as protein and DNA binding (Figure 6B and Table S7). Pathway enrichment analysis showed that upregulated genes for W5CC were enriched in 43 (level 1), 81 (level 2), and 85 (level 3) pathways (Table S8). The most significantly enriched pathways for level 1 genes included the metabolism of cofactors and vitamins (one carbon pool by folate), signal transduction (Hippo signaling pathway), and human disease (such as colorectal, endometrial, and gastric cancer) (Figure 7B). On the other hand, the W5CC downregulated genes were assigned to 347 (level 1), 382 (level 2), and 636 (level 3) GO processes (Table S7). Similar to the W1CC result, defense response to virus, adhesion of symbiont to host, immune system process, and lipid metabolic process in biological processes were significantly enriched for downregulated level 1 genes of W5CC (Figure 6B). A total of 41 (level 1), 73 (level 2), and 99 (level 3) pathways were significantly enriched for W5CC downregulated genes, containing immune system (NOD-like receptor signaling pathway), transport and catabolism (peroxisome, lysosome, and phagosome), viral infectious diseases (such as measles and hepatitis C), and multiple metabolism processes (such as xenobiotics by cytochrome P450 metabolism, drug metabolism, and glycosphingolipid biosynthesis) (Figure 7B and Table S8).

### 2.8. Validation of the RNA-Seq Results by qRT-PCR and ELISA

**Figure 8:**
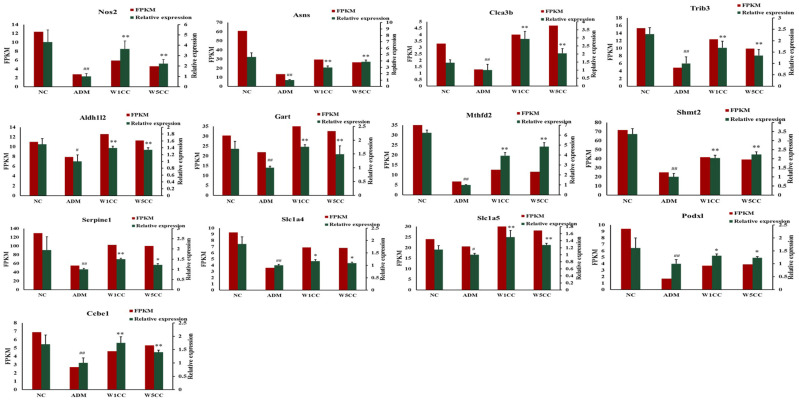
Validation of RNA-seq results with qRT-PCR. Confirmation of expression patterns for 13 genes involved in inflammation, metabolism of amino acids, cell proliferation and differentiation, DNA synthesis and damage repair, and folic acid biosynthesis. FPKM values from the RNA-seq are shown in red column; qRT-PCR values are shown in the green column. Data are presented as mean ± SD (n = 3). ^#^ indicates *p* < 0.05 or ^##^ indicates *p* < 0.01 as compared with NC group; * indicates *p* < 0.05 or ** indicates *p* < 0.01 as compared with ADM group.

**Figure 9:**
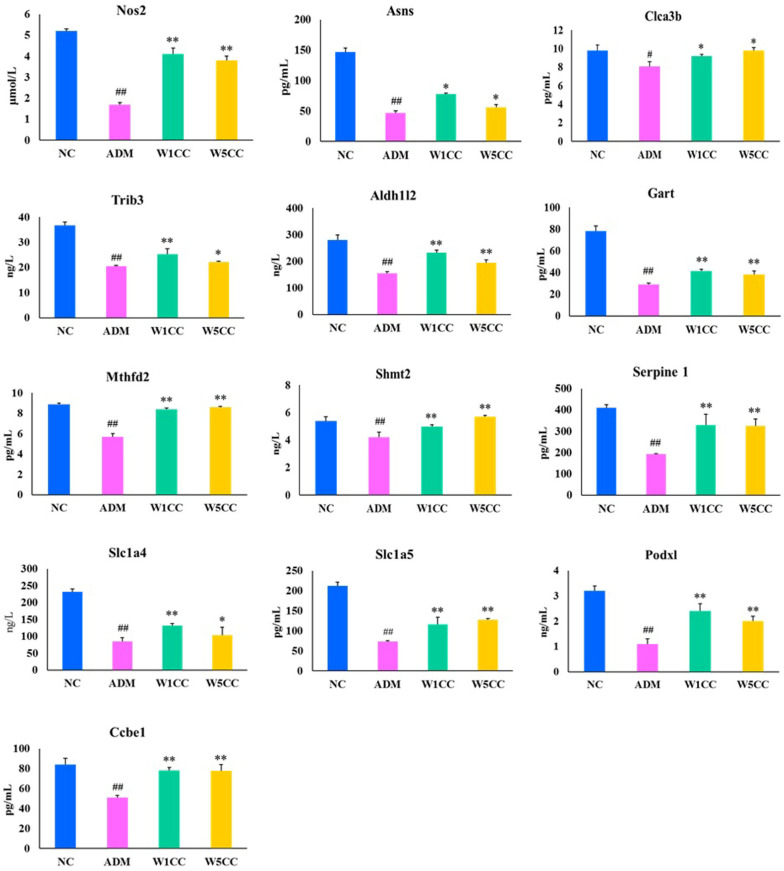
Validation of RNA-seq results with gene protein expression. Confirmation of expression patterns for 13 genes involved in inflammation, metabolism of amino acids, cell proliferation and differentiation, DNA synthesis and damage repair, and folic acid biosynthesis. Data are presented as mean ± SD (n = 3). ^#^ indicates *p* < 0.05 or ^##^ indicates *p* < 0.01 as compared with NC group; * indicates *p* < 0.05 or ** indicates *p* < 0.01 as compared with ADM group.

To validate the veracity and reliability of the DEGs identified by RNA-seq, 13 genes related to inflammation, metabolism of amino acids, cell proliferation and differentiation, DNA synthesis and damage repair, and folic acid biosynthesis were selected for qRT-PCR validation based on fold change and functional enrichment results. The qRT-PCR data were significantly correlated with the RNA-seq results (Figure 8), demonstrating the credibility of the transcriptome results. We further validated the veracity and reliability of RNA-seq by detecting the protein expression of these 13 genes through ELISA analysis. The ELISA analysis data were significantly correlated with qRT-PCR data and RNA-seq results (Figure 9).

## 3. Discussion

Podocytes, as terminally differentiated epithelial cells, cover the outer surface of glomerular capillaries and form the glomerular filtration barrier in along with the GBM and glomerular endothelial cells [27]. Podocytes live under various stresses and pathological stimuli. They adapt to maintain homeostasis, but excessive stress leads to maladaptation with complex biological changes including loss of integrity and dysregulation of cellular metabolism and result in podocytopathies [28]. Podocytopathies are renal diseases in which directly or indirectly drive proteinuria or nephrotic syndrome. The pathogenesis of nephrotic syndrome is recognized as the dedifferentiation of podocytes that causes damage to the actin cytoskeleton and/or the slit diaphragms (SDs), and podocytes’ intercellular junctions. Evidence has shown that podocytes are the center of pathophysiology and targets of therapy in nephrotic syndrome [29,30]. The lack of effective interventions on preventing podocyte injury demands a better understanding of the key and universal molecules involved in various podocytopathies, which may provide potential diagnostic and therapeutic measures for patients with proteinuric renal disease [31].

**Figure 10:**
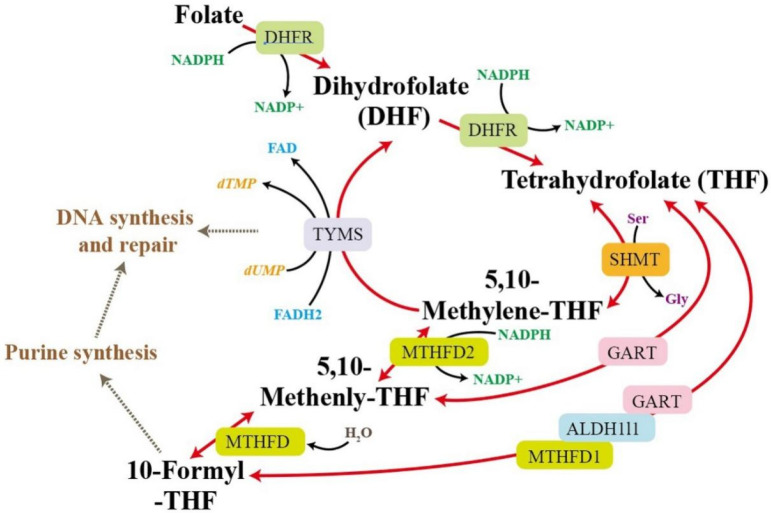
The process of one-carbon pool metabolic pathway regulated by folate.

**Figure 11:**
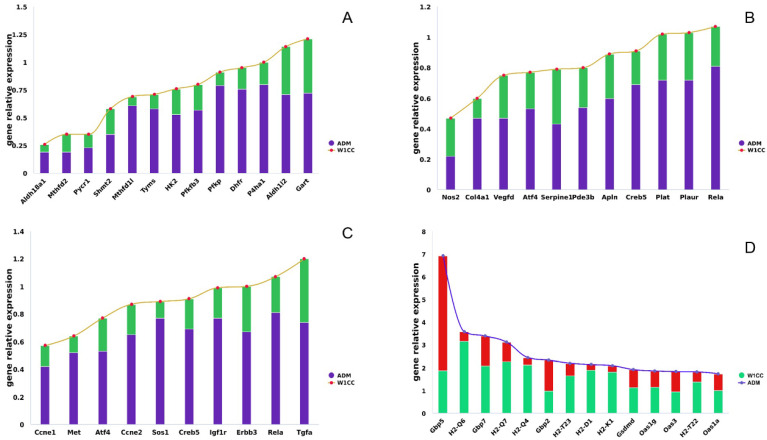
The genes potentially participated in alleviating mpc5 cell injury after W1CC treatment. The Y-axis represents the relative expression value of genes, “1” represented the normal expression value. ((**A**): upregulated genes involved in the metabolism pathway; (**B**): upregulated genes involved in the relaxin signaling pathway, apelin signaling pathway, and complement and coagulation cascades pathway; (**C**): upregulated genes that are involved in the PI3K-Akt, MAPK, TNF, and ErbB signaling pathways; (**D**): downregulated genes involved in the NOD-like receptor signaling pathway and immune systems).

The exact mechanism of WCCs in the treatment of nephrotic syndrome is still unclear. We conducted high-throughput screening of its efficacy using transcriptomics and validated the transcription levels of several differentially expressed genes. Under the treatment of W1CC, the upregulated genes were enriched in multiple metabolism pathways, such as one-carbon pool by folate, fructose, and mannose metabolism, and glycolysis, arginine, and proline metabolism. Enhanced expression of called-back genes is most prominent in the one-carbon pool by folate pathway, including *Mthfd2*, *Dhfr*, *Aldh1l2*, *Mthfd1l*, *Tyms*, *Gart*, and *Shmt2*. The one-carbon pool refers to a group of metabolites involved in various cellular processes that require one-carbon units, such as nucleotide synthesis, methylation reactions, and amino acid metabolism. Intermediate product 5,10-Metheny-THF within the pathway can be converted into dihydrofolate (DHF) by TYMS, meanwhile releasing methyl groups, which are needed for the conversion of deoxyuridine monophosphate (dUMP) to deoxythymidine monophosphate (dTMP), a precursor for thymine in DNA [32]. The one-carbon units released from this pathway are required for the nucleotide building blocks (thymidine and purines) that constitute DNA and participate in repairing damaged DNA, ensuring the preservation of genome integrity and stability [33,34]. SHMT facilitates the conversion of serine to glycine by transferring the hydroxymethyl group (-CH_2_OH) from serine to tetrahydrofolate (THF). This reaction is necessary for synthesizing glycine, an essential amino acid involved in protein synthesis and several metabolic pathways [35]. Furthermore, the one-carbon pool synthesizes coenzymes such as THF and S-adenosylmethionine (SAM). THF functions as a carrier of one-carbon units, while SAM provides the methyl groups for methylation reactions [36]. The one-carbon pool by folate pathway is a fundamental metabolic pathway that provides one-carbon units for critical processes, essential for maintaining normal cellular function and overall health (Figure 10). After being induced by ADM, the expression levels of seven core genes in this pathway (*Mthfd2*, *Dhfr*, *Aldh1l2*, *Mthfd1l*, *Tyms*, *Gart*, and *Shmt2*) were significantly suppressed, whereas W1CC treatment led to their upregulation (Figure 11A). This suggests that W1CC might alleviate mpc5 cell injury by enhancing the expression of genes associated with the one-carbon pool by folate pathway, guaranteeing the availability of methyl groups and maintaining essential cellular functions, such as DNA synthesis and repair, methylation reactions, amino acids metabolism, and coenzyme synthesis.

In addition, *HK2*, *Pfkfb3*, and *Pfkp* in fructose and mannose metabolism mediate the D-fructose transformed into D-fructose-1,6-bisphosphate (FBP), an essential intermediate molecule in the metabolic pathway known as glycolysis [37]. Its primary function is to regulate and facilitate the breakdown of glucose, resulting in the production of energy in the form of ATP [38]. In the arginine and proline pathway, the called-back genes (*Aldh18a1*, *P4ha1*, and *Pycr1*) were mainly enriched in the module of 4-hydroxy-proline (HYP) biosynthesis. HYP is crucial for the proper formation, stability, and function of collagen, essential for maintaining the structural integrity of various tissues in the body [39].

The endocrine system was also impacted by ADM and W1CC treatment. The downstream genes in the relaxin signaling pathway (class RXFP1), including *Atf4*, *Creb5*, *Vegfd*, *Rela*, *Nos2*, and *Col4a1*, were upregulated after W1CC treatment (Figure 11B). Atf4 and Creb5 are transcription factors that bind to specific DNA sequences in the promoter region of target genes, including the *Vegf* gene [40,41]. Atf4 typically recognizes ATF/CRE (activating transcription factor/cAMP response element) sites, while CREB binds to cAMP response elements (CREs). Upon activation, ATF and CREB can enhance the transcriptional activity of the *Vegf* gene, leading to angiogenesis, endothelial cell proliferation, migration, and survival [42,43]. In addition, as a component of the NF-κB complex, *Rela* can directly bind to the promoter of the *Nos2* gene, augmenting its transcriptional activity [44]. This results in the production of second messenger nitric oxide (NO), leading to an increase in effective renal plasma flow [45], glomerular filtration rate (GFR) [46], and sodium excretion [47], while simultaneously reducing fibrosis [48]. Furthermore, *Col4al* is a gene that encodes the alpha-1 chain of type IV collagen, a major component of basement membranes [49]. Mutations in *Col4a1* have been associated with fibrosis in several organs [50]. Duan et al. identified *Col4a1* as the functional homolog of human *Col4a5* in the fly nephrocyte (equivalent of human podocyte). Fly nephrocytes deficient for *Col4a1* showed an irregular and thickened basement membrane and significantly reduced nephrocyte filtration function [51]. Taken together, W1CC can reverse the downregulated expression of the aforementioned genes in ADM-induced mpc5 cell back to near-normal levels, thereby contributing to cell proliferation, survival, and conferring resistance against fibrosis.

The apelin plays important roles in the physiology and pathophysiology of several organs, including the regulation of oxidative stress, angiogenesis, metabolic balance, cell proliferation, apoptosis, and inflammation [52,53]. In the apelin signaling pathway, the expression level of *Nos2*, *Pde3b*, *Apln*, *Serpine1*, and *Plat* was significantly downregulated in ADM-induced mpc5 cells and exhibited near-normal levels under the treatment of W1CC (Figure 11B). *Apln*, *Serpine1*, and *Plat* are involved in cardiovascular regulation. APLN and Surpine1 are peptide hormones that regulate cardiovascular function and have opposing effects on vascular tone, with *Apln* acting as a vasodilator and *Serpine* as a vasoconstrictor [54,55]. The gene expression and balance between the actions of *Apln* and *Surpine* help maintain vascular tone and blood pressure homeostasis. In contrast, Plat, an enzyme involved in the dissolution of blood clots, promotes fibrinolysis and aids in the restoration of blood flow, thereby preventing the formation of excessive or harmful clots and reducing the risk of cardiac fibrosis [56]. Primary nephrotic syndrome is involved in the abnormality of circadian blood pressure [57]. The W1CC appeared to have the capacity to preserve cardiovascular health by regulating the expression levels of these genes to stabilize blood pressure and forestall cardiac fibrosis.

The PI3K-Akt signaling pathway is activated by many types of cellular stimuli or toxic insults and regulates fundamental cellular functions such as transcription, translation, proliferation, growth, and survival [58]. Several genes in this pathway involved in cell cycle progression (*Ccne1* and *Ccne2*) [59], cell proliferation (*Tgfa*, *Met*, *Erbb3*, *Igf1r*, and *Sos1*) [60,61,62,63,64], and cell survival (*Atf4*, *Creb5,* and *Rela*) [65,66,67] were significantly suppressed by ADM and rebound expression after W1CC treatment (Figure 11C). These upregulated genes were shared in multiple signaling pathways, such as MAPK, TNF, and ErbB signaling pathways, thereby underscoring the crucial role of maintaining these gene expressions.

In the NOD-like receptor signaling pathway, the gene expression of *Gbp* (*Gbp2*, *Gbp5*, and *Gbp7*) and *Gsdmd* was significantly upregulated by ADM and returned to near-normal level by W1CC treatment (Figure 11D). GBPs participate in targeting and disrupting pathogen-containing vacuoles (especially binding Gram-negative bacteria), thereby facilitating pathogen exposure to host defense mechanisms [68]. Subsequently, Gsdmd triggers pyroptosis (a form of programmed cell death) by forming pores in the cell membrane [69]. Excessive or dysregulated pyroptosis in sterile podocyte may contribute to cell damage and disease progression. In addition, the expression pattern of *Oas1a*, *Oas1g*, and *Oas3* in the NOD-like receptor signaling pathway exhibited similarities with those of *GBP* and *Gsdmd*, as previously mentioned. *Oas1a*, *Oas1g*, and *Oas3* play a crucial role in the innate immune response against viral infection, leading to viral RNA degradation and replication inhibition [70,71,72]. However, overexpression of these genes in virus-free podocytes can have broader effects on cellular processes, potentially influencing cell proliferation, apoptosis, and homeostasis. Furthermore, several histocompatibility 2 (*H2*) in other immune systems, such as natural killer cell mediated cytotoxicity (*H2-K1*, *H2-D1*, and *H2-T23*) and antigen processing and presentation (*H2-K1*, *H2-D1*, *H2-T23*, *H2-Q4*, *H2-Q6*, *H2-T22*, and *H2-Q7*), were upregulated by ADM and returned to near-normal levels by W1CC treatment (Figure 11D). Overexpression of the *H2* gene could potentially disrupt the immune tolerance mechanism, resulting in the activation of autoreactive immune cells and the onset of autoimmune diseases [73]. Conversely, the expression of *Plat* and *Plaur* in the complement and coagulation cascades pathway was suppressed by ADM treatment and reversed following W1CC treatment (Figure 11B). The restoration of *Plat* and *Plaur* expression is instrumental in cell adhesion, migration, and proliferation. These findings suggested that W1CC exhibited the ability to regulate genes associated with specific immune pathways, thereby inhibiting excessive immune responses and the development of immune tolerance and related diseases, as well as maintaining cell adhesion, migration, and proliferation.

**Figure 12:**
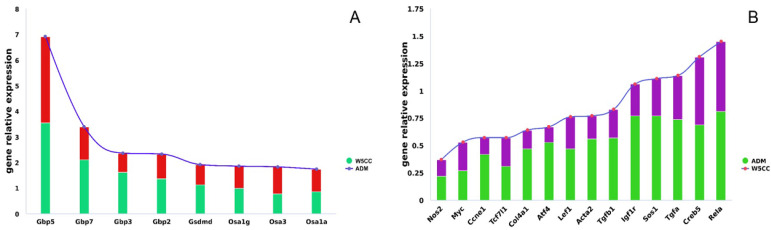
The genes potentially participated in alleviating podocyte injury after W5CC treatment. The Y-axis represents the relative expression value of genes; “1” represents the normal expression value. ((**A**): upregulated genes that involve in relaxin, PI3K-AKT, MAPK, and TNF pathways; human disease pathways; and Hippo and Wnt signaling pathways; (**B**): downregulated genes that are involved in the NOD-like receptor signaling pathway).

Regarding the upregulated genes of W5CC, the enriched metabolic pathways were limited, only encompassing one carbon pool by folate, as well as arginine and proline metabolism. These two pathways were concurrent with W1CC but exhibited fewer upregulated genes in W5CC. On the other hand, 12 metabolic pathways (including modules within the pathway) enriched with downregulated genes were found to be shared with W1CC, including amino acids metabolism (such as valine, leucine, and isoleucine degradation), carbohydrate metabolism (such as galactose metabolism), lipid metabolism (fatty acid degradation), and xenobiotic biodegradation and metabolism (such as drug metabolism). Similar to W1CC effects, the downregulated genes of W5CC were also enriched in the immune system of the NOD-like receptor signaling pathway. The expression of *Gbp2*, *Gbp3*, *Gbp5*, *Gbp7*, *Gsdmd*, *Oas1a*, *Oas1g*, and *Oas3* of mpc5 cell injury by ADM were returned to near-normal levels after W5CC treatment (Figure 12A). The normal expression of these genes is capable of inhibiting cell pyroptosis and damage, maintaining cell homeostasis, thereby enhancing the cell survival rate. Moreover, the upregulated genes were enriched in the RXFP1 module in the relaxin signal pathway, paralleling the W1CC effects. The genes *Creb5*, *Atf4*, *Rela*, *Nos2*, *Acta2*, *Tgfb1*, and *Col4a1*, which are involved in anti-fibrosis [74,75,76,77,78,79,80], were suppressed in ADM-induced injury mpc5 cell but returned to near-normal expression following W5CC treatment (Figure 12B). Furthermore, a substantial number of the upregulated genes in W5CC that enriched in PI3K-AKT, MAPK, and TNF pathways were also shared with W1CC, including the module of cell cycle progression (*Myc* and *Ccne1*) [81,82], cell proliferation (*Tgfa*, *Igf1r*, and *Sos1*) [64,83,84], and cell survival (*Atf4*, *Creb5,* and *Rela*) [65,66,67]. The findings suggest that W1CC and W5CC exhibited several comparable protective effects against ADM-induced injury on mpc5 cells.

Unlike the results from W1CC, the significantly enriched pathways of upregulated genes in W5CC were primarily associated with human disease pathways. Among these disease pathways, the upregulated genes were predominantly enriched within the cell proliferation module of cancer-associated pathways, including *Lef1*, *Tcf7l1*, and *Myc* genes. Lef1 and Tcf7l1 are transcription factors that can interact with each other and form complexes that bind to the DNA region of *Myc*, leading to the upregulation of *Myc* expression [85]. In essence, *Lef1*, *Tcf7l1*, and *Myc* also participate in Hippo and Wnt signaling pathways, and play crucial roles in various cellular processes, particularly in regulating cell proliferation, growth, differentiation, and metabolism [86,87,88,89]. Suppressing the expression of *Lef1*, *Tcf7l1*, and *Myc* can lead to cell cycle arrest, apoptosis, and the inhibition of cell proliferation and differentiation, while significantly overexpressing these genes may promote resistance to cell death and tumorigenesis [90,91,92]. *Lef1*, *Tcf7l1*, and *Myc* were significantly downregulated following ADM treatment, yet displayed a rebound effect after W5CC treatment, albeit without reaching their normal levels (Figure 12B). The present findings implied that W5CC partially restores the expression of these genes, thereby preserving normal cellular functions such as cell proliferation and differentiation. However, it may not trigger excessive expression and elicit cellular damage.

Although we have preliminarily discussed the protective mechanism of WCCs on mpc5 cell, we recognize that there are some limitations to this study. Firstly, our research is still at the in vitro level, indirectly constructing an in vitro model of mpc5 cell damage, which can only partially indicate changes in its biological function. Therefore, we still need to further construct animal models for in-depth exploration. Secondly, current research on molecular mechanisms is limited to the transcriptional level and has not delved into the protein level, and thus unable to explain the exact mechanism of WCCs. Therefore, in order to better explain the pharmacological mechanism of WCCs, more protein-level experimental studies are needed to reveal the effects of WCCs on protein, protein–protein, and signaling pathways. Finally, if we can conduct clinical randomized controlled trials and obtain patient samples to further validate the speculation and results of this experiment, then we can truly provide reference for the clinical treatment of nephrotic syndrome.

In conclusion, we found in this study that WCCs can improve ADM-induced mpc5 cell injury, while accompanied by significant changes in RNA expression in mpc5 cells. Based on the results of cell pharmacodynamic experiments and RNA-seq analysis, the molecular mechanism of WCCs for alleviating ADM-induced mpc5 cell injury is mainly probably through regulating cellular metabolism, steroid hormone biosynthesis, endocrine system, immune pathways, and human disease pathways, thus promoting mpc5 cell injury repair, proliferation, and growth, and inhibiting apoptosis. Polysaccharides and nucleoside were considered as the mainly bioactive ingredients in WCCs for alleviating mpc5 injury. These results provide useful cues for employing Chinese cordyceps as an effective method for the treatment of nephrotic syndrome.

## 4. Materials and Methods

### 4.1. Preparation of Sample Solutions

The Chinese cordyceps used in this present study were produced according to the described method in a previous study [93] and were then freeze dried and milled into powder through 0.25 mm sieve. In brief, dried and crushed CC (50 g) was extracted with water under reflux for 1 h and repeated three times. The proteins in the aqueous extract were removed according “Sevage method”. The ethanol was added to the aqueous extract until 20% concentration and precipitated at 4 °C for 12 h, producing sediment 1 (W1CC) after centrifugation at 1000× *g* for 5 min at room temperature. Then, ethanol was added to 40%, 60%, or 80% concentration, respectively, to obtain W2CC, W3CC, and W4CC. The remaining aqueous extract was regarded as W5CC. Then the sample solutions of WCCs (including W1CC, W2CC, W3CC, W4CC, and W5CC) were all prepared. The voucher specimen (ID-S-190110) was deposited in the herbarium of Institute of Zoology, Guangdong Academy of Sciences. The study of morphological and microscopic characteristics and phytochemical profile was described in our previous work [94].

### 4.2. Quantitative Determination of Polysaccharides of WCCs

The quantitative determination of polysaccharides in W1CC, W2CC, W3CC, W4CC, and W5CC were determined according to the phenol–sulfuric acid method. Briefly, glucose solutions of different concentrations (0, 0.02, 0.04, 0.06, 0.08, and 0.1 mg/mL) were used as the standard [95].

### 4.3. Identification of Nucleoside Ingredients in W5CC

A chromatographic column, Agilent InfinityLab Poroshell 120 EC-C18 (4.6 mm × 100 mm, 2.7 μm) (Agilent, Beijing, China), with the mobile phase of ultrapure water A and acetonitrile B (Guanghua, Guangzhou, China) (gradient: 0–10 min, 0% B; 10–25 min, 0–10% B; 25–30 min, 10–20% B; 30–35 min, 20–0% B; 35–40 min, 0% B) was used for HPLC (Agilent, Santa Clara, CA, USA) analysis. The flow velocity, detection wave length, and column temperature were 0.5 mL/min, 227 nm, and 30 °C, respectively. The major ingredients of W5CC were identified by comparing their retention time with the reference substance.

The electrospray ionization quadrupole time-of-flight mass spectrometer (ESI-Q-TOF-MS) (Thermo Scientific, Waltham, MA, USA) adopts positive (ESI+) ion mode for detection, with a mass range of 100–1000 Da. The optimized parameter settings for the mass spectrometer are as follows: capillary voltage of 2.3 kV, sample cone voltage of 25 V, and source and desolvation temperatures of 150 °C and 350 °C, respectively. The cone gas flow rate was 50 L/h, the desolvation gas flow rate was 800 L/h, and nitrogen and argon were used as conical and collision gases. Data were switched between the low-energy (4 V) and elevated energy (10–55 V) acquisition modes every 0.2 s.

### 4.4. Establishment of ADM-Induced Podocyte Injury Model

Aims to verify the protective effect against ADM-induced podocyte injury of WCCs, mpc5 cells (Procell, Wuhan, China) were used to carry out the activity assay experiments. The cells were cultured in DMEM medium (Thermo Fisher Scientific, Waltham, MA, USA) supplemented with 10% fetal bovine serum (ExCell Bio, Canberra, Australia) and 1% penicillin/streptomycin (Thermo Fisher Scientific, Waltham, MA, USA) in a 5% CO_2_ atmosphere at 37 °C. The medium was renewed every 2 days until the cells reached 90% confluence.

Cells were adjusted to a density of 5 × 10^3^ cells/mL and plated into sterile 96-well plates and allowed to attach for 24 h before exposure to varying concentrations (0 μg/mL, 0.1 μg/mL, 0.2 μg/mL, 0.3 μg/mL, 0.4 μg/mL, 0.5 μg/mL, and 0.6 μg/mL) of ADM (Sigma-Aldrich, Saint Louis, MO, USA). Cell enumeration assays were performed by using cell counting kit-8 after 24 h incubation (cck-8, Dojindo, Kumamoto Prefecture, Japan). Colorimetric absorbance was determined at 450 nm using a microplate reader (Molecular Devices, Santa Clara Valley, CA, USA). The morphology was observed under an inverted microscope (EVOS f1, Thermo Scientific, Waltham, MA, USA). The ADM concentration with a cell inhibition rate close to 50% (IC_50_) was selected to establish the ADM-induced podocyte injury model.

### 4.5. In Vitro Cytotoxicity of WCCs

To evaluate the effect of W1CC, W2CC, W3CC, W4CC, and W5CC on cell proliferation, we performed cells seeding on a 96-well culture plate at a density of 5 × 10^3^/well/100 μL and treated them with the indicated concentrations of W1CC, W2CC, W3CC, W4CC, and W5CC for 24 h. The relative cell viabilities were measured using cck-8 according to the manufacturer’s instructions. Colorimetric absorbance was determined at 450 nm using a microplate reader. Distilled water was used as the vehicle control. The cell was calculated using the following equation: cell viability (100%) = (OD_treatment_/OD_control_) × 100%.

### 4.6. Protective Efficacy of WCCs on ADM-Induced mpc5 Cell Injury

The mpc5 cell culture method was the same as above. After attachment, respectively, cells were incubated with ADM (0.2 μg/mL) and different concentrations (100, 300, and 500 μg/mL) of WCCs simultaneously for an additional 24 h. Finally, the calculation method of cell viability rate is the same as above. Optimal WCCs concentrations for use in the following experiments were determined based on preliminary results.

### 4.7. Total RNA Preparation, cDNA Library Construction, and mRNA Sequencing

After corresponding treatment, mpc5 cells were rinsed three times with ice-cold phosphate-buffered saline, and total RNA from the mpc5 cells was extracted using TRIzol RNA Isolation Reagents (Thermo Fisher Scientific) according to the manufacturer’s instructions. A Nanodrop ND-2000 and non-denaturing agarose gel electrophoresis were used to determine the RNA’s concentration, integrity, and quantity. Twelve cDNA libraries were constructed, including the normal control group (NC, without any treatment), model control group (ADM, treated with 0.2 μg/mL ADM), W1CC group (W1CC, treated with 0.2 μg/mL ADM and 500 μg/mL W1CC), and W5CC group (W5CC, treated with 0.2 μg/mL ADM and 500 μg/mL W5CC), with 3 replicates for each group; for the full uncropped gels and blots image, see Figure S1. The library quality was checked using an Agilent 2100 Bioanalyzer (Agilent, Santa Clara, CA, USA) and ABI StepOnePlus Real-time PCR System (ABI, Carlsbad, CA, USA). The Illumina HiSeq^TM^ 2000 platform (Illumina, San Diego, CA, USA) was used for sequencing.

### 4.8. Transcriptome Quality Control and Annotation

The reads that contained an adapter sequence, with more than 10% uncertain base pairs, and with low quality were removed by using the SOAPnuke package (1.5.2) [96]. The resulting clean reads were used to perform quality control, base composition, and quality distribution. Only clean reads with a balanced composition and high distribution of a high-quality base (sequencing quality value > 20) were kept. The remaining clean reads were mapped to the mouse genome (PRJNA20689) using HISAT (2.0.6) [97]. StringTie (v1.0.4) was used to reconstruct the transcripts, and the potentially novel transcripts were predicted by cufflinks (v2.2.1) and CPC (v0.9r2) [98,99]. All the novel transcripts were annotated against the NCBI non-redundant protein and SWISS-PROT databases using BlastX (2.0) (E-value < 1 × 10^−5^).

### 4.9. Differentially Expressed Gene Analysis

RSEM v1.2.31 (RNA-seq by expectation maximization) was implemented to estimate the abundance of transcripts and fragments per million mapped read (FPKM) to calculate the digital gene expression profile [100]. DEGs of the samples (ADM vs. NC, W1CC vs. ADM, W5CC vs. ADM, W1CC vs. NC, and W5CC vs. NC) were calculated using the DESeq package (v1.10.1) on R software (v3.2.3) [101]. Benjamini and Hochberg’s approach was used to adjust the *p*-values to control the FDR [102]. Genes in different samples with FDR < 0.05 and |log_2_FC| ≥ 0.5 were considered as DEGs in the samples.

To investigate the protective effect against ADM-induced mpc5 injury of W1CC and W5CC, called-back genes (level 1 and level 2) or specific altered genes (level 3) were identified. Level 1 genes were significantly down-/upregulated (|log_2_FC|_ADM vs. NC_ > 0.5, FDR < 0.05) after induction by ADM, and meanwhile significantly up-/downregulated after treatments (|log_2_FC|_W1CC/W5CC vs. ADM_ > 0.5, FDR < 0.05). Level 2 genes were significantly or slightly down-/upregulated after ADM induction (|log_2_FC|_ADM vs. NC_ > 0.1, FDR < 0.05), and simultaneously significantly or slightly up-/downregulated after treatments (|log_2_FC|_W1CC/W5CC vs. ADM_ > 0.1, FDR < 0.05). Level 3 genes were unaltered after ADM induction (FDR > 0.05), but significantly or slightly up-/downregulated (|log_2_FC|_W1CC/W5CC vs. ADM_ > 0.1, FDR < 0.05) after treatments. The genes of levels 1 and 2 that exhibited downregulation (compared with NC) following ADM induction, but upregulation (compared with ADM) after treatments were denoted as up-called-back genes, and those genes displaying the inverse expression pattern were classified as down-called-back genes.

### 4.10. Functional Enrichment Analysis

Enrichment analysis was carried out for the gene list of DEGs based on the algorithm presented by GO::TermFinder (v0.86) on Perl script, *p*-values were Bonferroni-corrected [103], and the threshold for corrected *p*-values was set to ≤0.05. The pathway enrichment analysis of DEGs was conducted by KOBAS 3.0 with a threshold *p*-value set to ≤ 0.05 [104].

### 4.11. Confirmation of RNA-Seq Results with qRT-PCR Analysis and ELISA

RNA extracted from the treatments (W1CC and W5CC) or control group (NC, ADM) with three replicates for each group was used to validate the RNA-seq experiments. Total RNA was extracted by EASY spin tissue/Cell RNA rapid extraction kit (Aidlai Biotechnology, Beijing, China). The concentration, integrity, and quality of total RNA were detected by One Drop^TM^ spectrophotometer (Implen, Munich, Germany) and non-denaturing agarose gel electrophoresis. A total of 1 μg RNA from the transcriptome sample was used for cDNA synthesis, according to the manufacturer’s protocol (All-in-One First-Stand Synthesis MasterMix with dsDNase) (Xinkailai Biotechnology, Guangzhou, China). The 10 μL reaction system consisted of 1 μL of diluted cDNA (1:10), 5 μL SYBR Green qPCR Premix (Universal) (Kermey, Henan, China), 3.6 μL RNase-free water, and 0.2 μL of each primer. All reactions were performed on a CFX Connect^TM^ Real-Time System (BIO-RAD, Shanghai, China). Thermal cycling conditions were set to 95 °C for 30 s of initial denaturation, followed by 40 cycles of 95 °C for 10 s and 60 °C for 30 s of amplification. After the reaction, a melting curve analysis was applied to ensure the consistency and specificity of the amplified product. Three biological replicates were performed for each qRT-PCR amplification. Quantitative measurements were normalized by the reference gene *Rpl13a*. For the 13 DEGs and corresponding primers for qRT-PCR, see Table 2. The relative expression levels were calculated using the 2^−ΔΔCt^ method [105].

After corresponding treatment, mpc5 cells were rinsed three times with ice-cold phosphate-buffered saline, and total protein from the mpc5 cells was extracted according to the ELISA kit (Jiangsu Meimian, Yancheng, China) manufacturer’s instructions.

## 5. Conclusions

All statistical analyses were performed using the GraphPad Prism software 8.0 program. Measurement data are presented as mean ± standard deviation. Normally distributed data were compared between multiple groups using one-way analysis of variance (ANOVA). * *p* < 0.05, ** *p* < 0.01.

## Figures and Tables

**Table 1:** Summary of RNA-seq.

Sample Name	Total Reads	Clean Reads	Clean Bases (bp)	CleanGC (%)	CleanQ20 (%)	CleanQ30 (%)	Total Mapped
NC-1	43,857,918	43,982,656	6,597,398,400	50.57;50.83	97.63;96.38	93.71;90.65	96.06%
NC-2	52,689,236	52,742,126	7,911,318,900	50.34;50.30	97.93;96.74	94.38;91.70	96.67%
NC-3	39,438,362	39,522,550	5,928,382,500	51.04;51.17	97.72;97.25	93.91;92.66	96.50%
ADM-1	49,934,498	49,970,138	7,495,520,700	50.39;50.34	98.16;97.54	94.87;93.36	96.70%
ADM-2	44,982,290	45,062,288	6,759,343,200	50.10;50.34	97.51;96.96	93.33;91.96	96.16%
ADM-3	40,773,188	40,856,468	6,128,470,200	51.19;51.40	97.70;95.72	93.84;89.20	95.84%
W1CC-1	48,806,762	48,885,248	7,332,787,200	50.37;50.32	97.78;97.23	94.13;92.94	96.20%
W1CC-2	39,302,792	39,379,476	5,906,921,400	50.60;50.80	97.59;97.14	93.58;92.40	96.26%
W1CC-3	49,761,814	49,850,608	7,477,591,200	50.85;50.84	97.89;96.55	94.26;91.23	96.40%
W5CC-1	40,728,338	40,764,416	6,114,662,400	50.06;49.95	97.63;97.14	93.79;93.06	96.19%
W5CC-2	43,624,828	43,707,238	6,556,085,700	50.71;50.94	97.67;96.05	93.78;89.97	95.84%
W5CC-3	40,124,848	40,188,070	6,028,210,500	51.00;51.25	97.59;96.18	93.58;90.31	95.93%

**Table 2:** Primer sequences used for qRT-PCR analysis.

Gene Name	Forward Primer (5′-3′)	Reverse Primer (3′-5′)
*Nos2*	CCTGCTTTGTGCGAAGTGTC	CCCTTTGTGCTGGGAGTCAT
*Slc1a4*	GGCGAGACCAACGGCTAC	GATTTGCCCATCGCCGTCT
*Slc1a5*	TTCGCTATCGTCTTTGGTGTG	ATGGTGGCATCATTGAAGGAG
*Asns*	GACTGCAACCTGCTACCCAA	AAGGGAAACTTCTGGGAGGC
*Clca3b*	CCACCACACTCCCAGTCATC	AACAGGCAAAAACCCTTGGC
*Trib3*	ACCTTCAGAGCGACTTGTGG	TCTCCCTTCGGTCAGACTGT
*Podxl*	CTCTCCCATCTGGGCCTTTG	ACACAGCAGTTCCACGAGTT
*Aldh1l2*	CTAGAGGCGGGAACGGTTTT	TTCAGGGCCTCCTCACCTAA
*Gart*	TCACTGCTGCCCATCATACG	CACGAGAAGACCTTGGGGAC
*Mthfd2*	TATCACTCCCGTCCCTGGTG	GCGCTGTTTGGACTTGAACA
*Shmt2*	GTACGAACCGTAGACCCCAAG	CCCTGTAGGGATGGGAACAC
*Ccbe1*	GAAACAGAAGATGGCGCTGC	TGGAAGGTAGGCGTTTGAGG
*Serpine1*	TCTTTCCGACCAAGAGCAGC	AAAGGCTGTGGAGGAAGACG

## Data Availability

The data that support the findings of this study are openly available in Bioproject at URL https://submit.ncbi.nlm.nih.gov/subs/sra/SUB14006057/overview (accessed on 29 November 2023), reference number PRJNA1047188. All data generated or analyzed during this study are included in this article.

## Supplementary Materials

The following supporting information can be downloaded at: https://www.mdpi.com/article/10.3390/ijms251910352/s1.
