# Supplementary material for: RNA-Seq-Based Transcriptome Analysis of Chinese Cordyceps Aqueous Extracts Protective Effect against Adriamycin-Induced mpc5 Cell Injury
Source: Int J Mol Sci. 2024 Sep 26;25(19):10352. doi: 10.3390/ijms251910352 (PMC11476491; doi:10.3390/ijms251910352)
Supplement: Supplementary file 1 [file ijms-25-10352-s001.zip › supplementary materials/Figure S1.pdf]

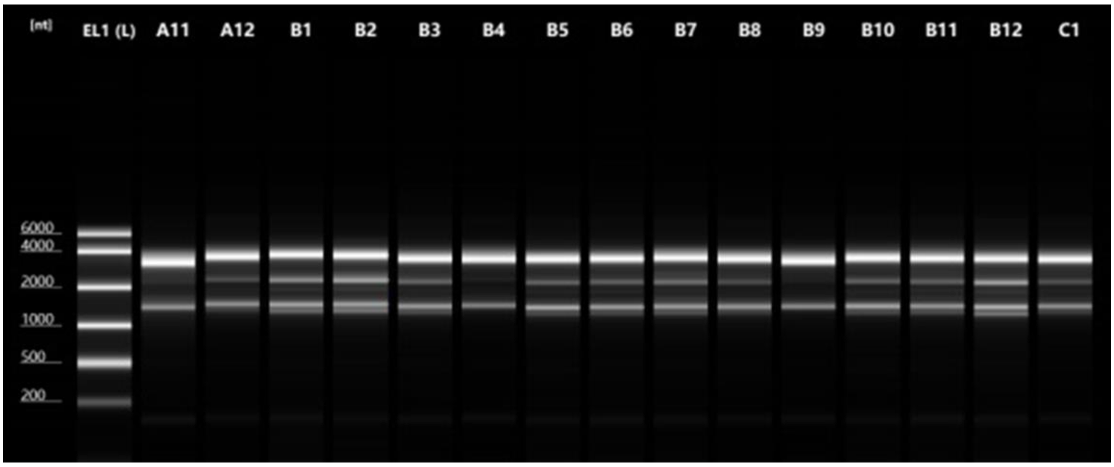

Figure S1. Gels and Blots image (A11, B4, B9: NC group; A12, B5, B10: ADM group; B1, B6, B11: W1CC group; B2, B7, B12: W5CC group; B3, B8, C1: Chinese cordyceps n-hexane extract group, which not mentioned in the manuscript.)
