# Supplementary figures and images for: RNA-Seq-Based Transcriptome Analysis of Chinese Cordyceps Aqueous Extracts Protective Effect against Adriamycin-Induced mpc5 Cell Injury
Source: Int J Mol Sci. 2024 Sep 26;25(19):10352. doi: 10.3390/ijms251910352 (PMC11476491; doi:10.3390/ijms251910352)

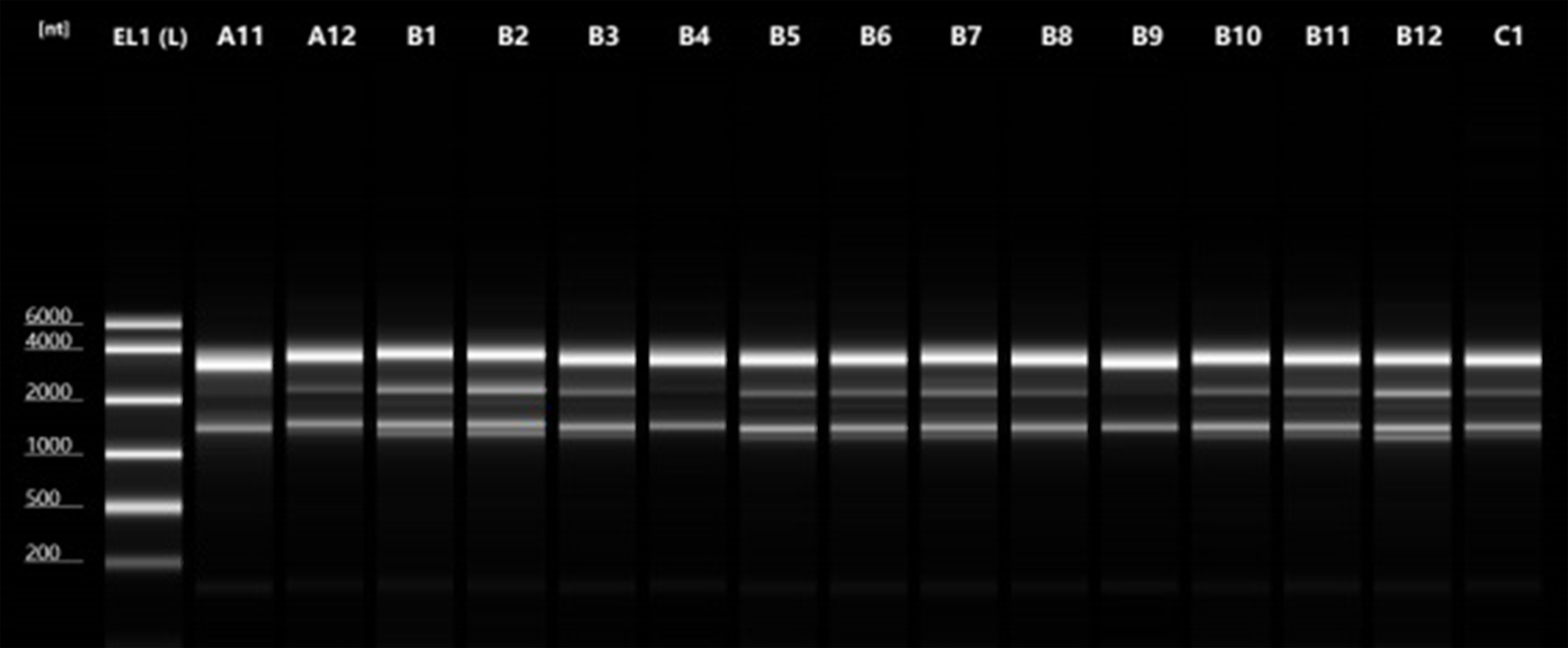

Supplement: Supplementary file 1 [file ijms-25-10352-s001.zip › supplementary materials/Figure S1.tif]
